# Side chain modified peptide nucleic acids (PNA) for knock-down of *six3* in medaka embryos

**DOI:** 10.1186/1472-6750-12-50

**Published:** 2012-08-17

**Authors:** Sebastian Dorn, Narges Aghaallaei, Gerlinde Jung, Baubak Bajoghli, Birgit Werner, Holger Bock, Thomas Lindhorst, Thomas Czerny

**Affiliations:** 1 Department for Biomedical Sciences, University of Veterinary Medicine, Veterinärplatz 1, A-1210, Vienna, Austria; 2 Department for Applied Life Sciences, University of Applied Sciences, FH Campus Wien, Helmut-Qualtinger-Gasse 2, A-1030, Vienna, Austria; 3 Current address: Director’s Research Unit, European Molecular Biology Laboratory (EMBL), Meyerhofstrasse 1, 69117, Heidelberg, Germany; 4 Ugichem GmbH, Mitterweg 24, A-6020, Innsbruck, Austria

**Keywords:** PNA, Knock down, Medaka, Six3

## Abstract

**Background:**

Synthetic antisense molecules have an enormous potential for therapeutic applications in humans. The major aim of such strategies is to specifically interfere with gene function, thus modulating cellular pathways according to the therapeutic demands. Among the molecules which can block mRNA function in a sequence specific manner are peptide nucleic acids (PNA). They are highly stable and efficiently and selectively interact with RNA. However, some properties of non-modified aminoethyl glycine PNAs (aegPNA) hamper their *in vivo* applications.

**Results:**

We generated new backbone modifications of PNAs, which exhibit more hydrophilic properties. When we examined the activity and specificity of these novel phosphonic ester PNAs (pePNA) molecules in medaka (*Oryzias latipes*) embryos, high solubility and selective binding to mRNA was observed. In particular, mixing of the novel components with aegPNA components resulted in mixed PNAs with superior properties. Injection of mixed PNAs directed against the medaka *six3* gene, which is important for eye and brain development, resulted in specific *six3* phenotypes.

**Conclusions:**

PNAs are well established as powerful antisense molecules. Modification of the backbone with phosphonic ester side chains further improves their properties and allows the efficient knock down of a single gene in fish embryos.

## Background

Antisense oligonucleotides are designed for sequence specific binding of complementary regions on their target mRNA. The binding either results in mRNA cleavage, caused by the activation of endonucleases RNase H or L [1], or in the inhibition of translation [2]. Different modifications of the nucleic bases or the backbone were introduced to improve their activity and biological stability. In morpholino antisense molecules, the ribose is replaced by morpholino rings and non-ionic phosphorodiamidate is used instead of phosphodiester linkages [3]. Their binding strength closely resembles that of RNA or DNA molecules, therefore, molecules with a length of 25 nucleotides are used for efficient transcriptional blockage. For translational blocking the 5’ untranslated region or the region around the start codon of the target mRNA are selected [4]. Morpholino oligomers can also be used for efficient blocking of the splice machinery [5]. The highly specific effect of morpholino oligomers on gene silencing could be demonstrated in sea urchins [6], Xenopus [7], Zebrafish [8] and Medaka [9]. Today morpholino oligos represent the gold standard for gene specific knock down in many species.

In 1991 Nielsen and colleagues created peptide nucleic acids (PNA) which instead of the phosphate ribose ring of DNA contain a polyamide backbone of N-(2-aminoethyl)-glycine (aeg) units [10]. aegPNAs bind to complementary RNA or DNA in a sequence-specific manner [11,12]. The chemical structure is responsible for a high stability against proteases, nucleases as well as thermal and pH fluctuations [13]. The entire neutral charge of the molecule decreases the electrostatic repulsion, which results in high hybridization affinity with RNA and DNA. Consequently short probe lengths (13–18 bases) are sufficient for selective binding, thereby reducing the probability for forming secondary structures [13-15]. In addition, the introduction of mismatches has a stronger effect on the stability of PNA/DNA interactions in comparison to DNA/DNA duplexes, demonstrating the high specificity of PNAs [16]. Several *in vitro* techniques make use of the extraordinary affinity of PNAs [17,18]. *In vivo* techniques also strongly benefit from the highly specific binding to mRNAs, however, so far unmodified aegPNAs have not been successful in injection experiments for gene specific knock down in animal models [19]. One problem for the application of PNAs is the low solubility due to the absence of charges. Introduction of negative charges by forming hetero-oligomers of alternating trans-4-hydroxy-L-proline/phosphonate polyamides with DNA bases (HypNA-pPNA) allowed specific down regulation of target genes in zebrafish embryos [20]. In addition, various end-modifications of PNAs have been developed, which mainly address the improvement of their cell delivery [21,22].

Here we tested the modification of the PNA backbone in order to keep the conformation most similar to the well established original aegPNAs. The resulting pePNAs contain phosphonic ester side chains in an otherwise non-modified polyamide backbone. The neutral pePNAs are highly soluble, but keep the high affinity and specificity of aegPNAs. In particular mixed versions of pe- and aegPNA components show favourable properties. We demonstrate the efficiency of these new antisense molecules by blocking gfp expression and *in vivo* down regulation of *six3* gene function in medaka embryos.

## Results and discussion

### Synthesis of novel peptide nucleic acids

**Figure 1:**
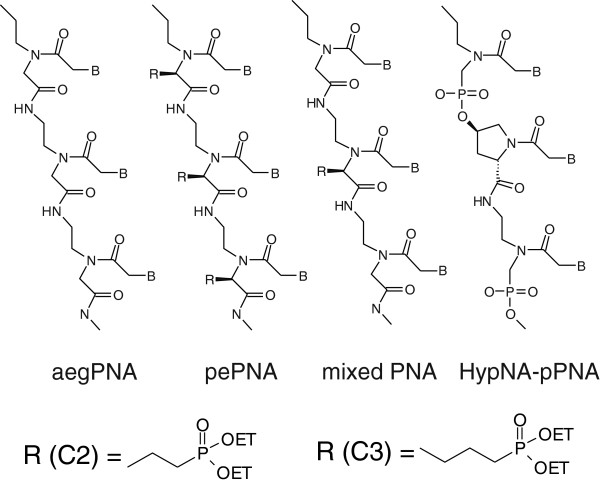
**Chemical structure of PNAs.** Schematic structure of aegPNAs, pePNAs, mixed PNAs (containing both aeg- and pePNA monomers) and HypNA-pPNAs is shown. pePNAs contain C2-phosphonoester residues, mixed PNAs contain C3-residues.

aegPNAs bind corresponding RNA sequences with high affinity and specificity, however, unfavourable properties like their low solubility hamper their application *in vivo*. By employing the building blocks shown in Additional file 1: Figure S1, we introduced phosphonic ester side chains into the otherwise non-modified backbone of aegPNAs. As a result novel phosphonic ester PNA variants (pePNA) were obtained (see Figure 1).

First we investigated the properties of a 15mer pePNA. The oligomer exhibited good solubility in water and we were able to prepare highly concentrated stock solutions (10 mM). However, it turned out that pePNAs capped with acetyl at the C-terminus tend to form stable foam after sonification at this concentration. A possible explanation might be the large number of phosphonic esters residues, which introduce a highly polar but not ionic character into the oligomer. The tendency to form foams was not observed when the pePNAs were permanently charged with trimethyl-lysine (TML) at the C-terminus prior to capping with acetyl (see Additional file 1: Figure S1).

### pePNAs efficiently block translation of *gfp* mRNA

**Figure 2:**
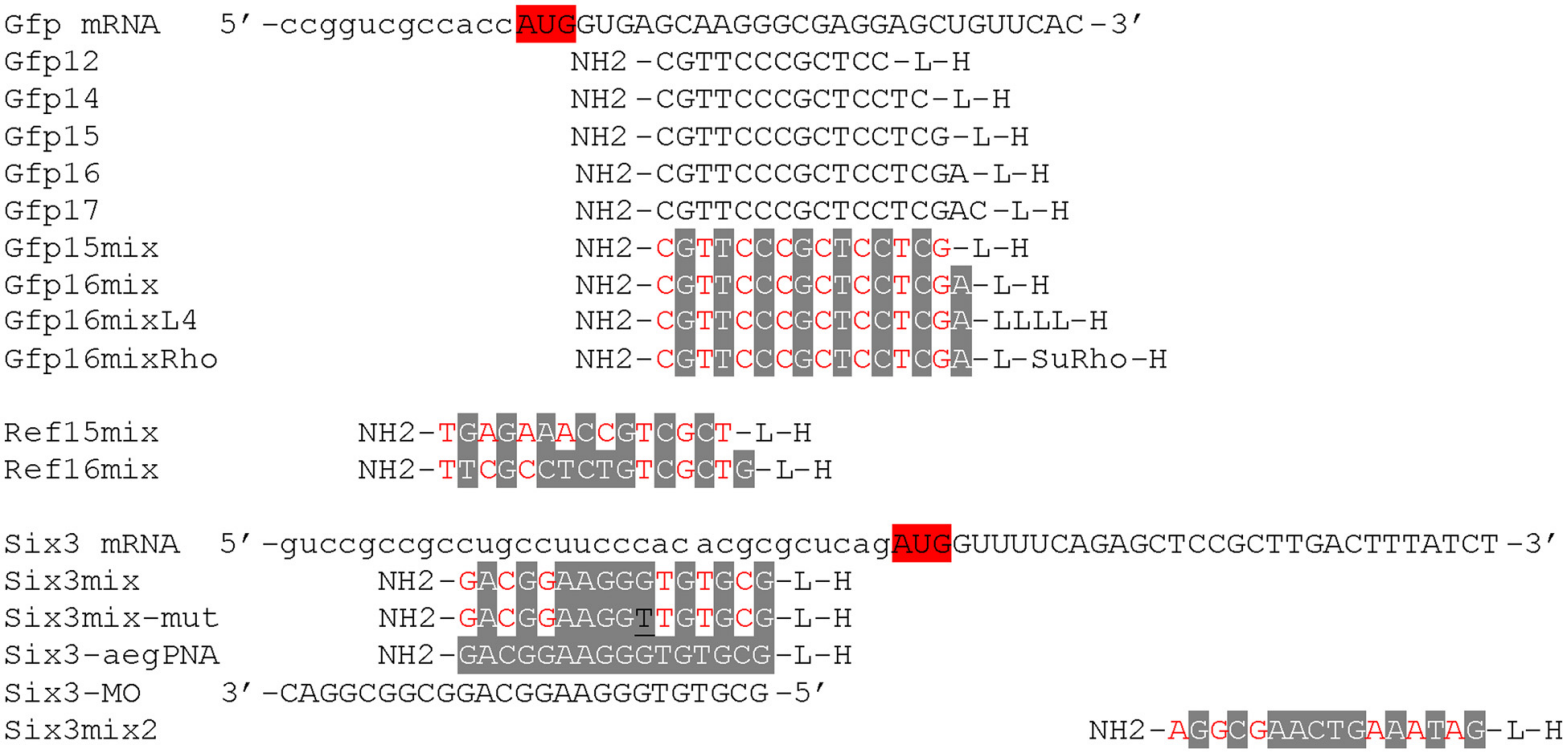
**Sequences of the antisense molecules and their targets.** The mRNA targets are shown in the 5’-3’ orientation, capital letters indicate the coding region. The AUG start codon is marked by red overlay. A morpholino oligo is indicated by MO (Six3-MO). All other sequences represent PNAs, pePNA-C2 components are shown in black, pePNA-C3 components in red and aegPNAs components are marked by gray overlay. Underlined bases (black) represent mismatches. All PNA and morpholino oligos are shown in the 3’-5’ (NH2-H for PNAs) orientation. L means trimethyl-lysine and LLLL a combination of 4 such residues. SuRho indicates sulforhodamine B.

**Figure 3:**
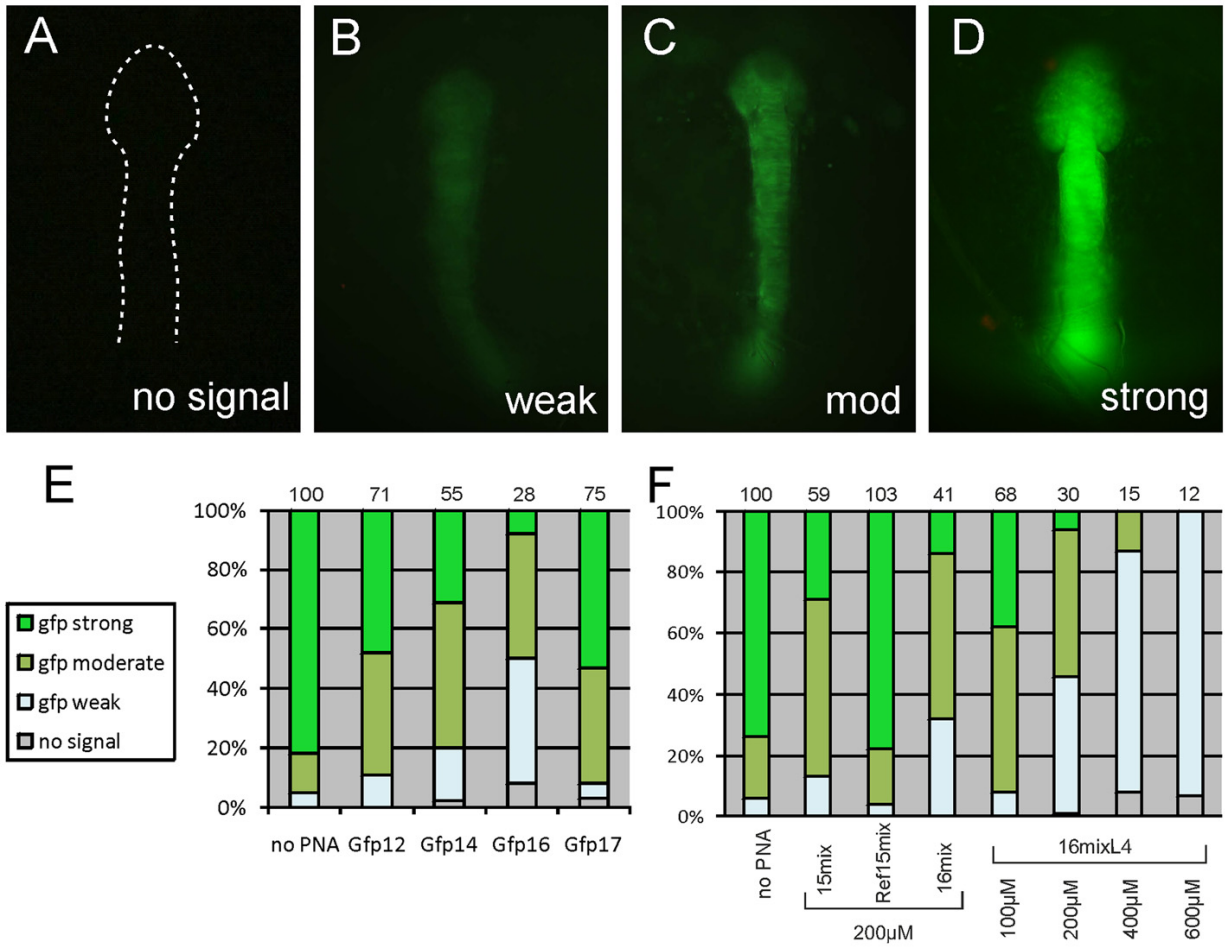
**Translational blocking of pePNAs after injection of gfp mRNA into medaka embryos.** The pePNAs were coinjected with gfp mRNA into medaka embryos at the one cell stage. 24 hours later the intensity of the gfp fluorescence was qualified as weak (**B**), moderate (**C**; mod) or strong (**D**). (**A**) Shows an uninjected embryo (no signal, corresponding to a complete knock down of gfp). The embryos are shown in a dorsal view, anterior to the top. A graph summarizing the experiments for optimisation of the PNA length is shown in (**E**). The names of the PNAs are explained in Figure 2. The embryos were injected with a mixture of 10 ng/μl gfp mRNA and 200 μM PNAs preincubated for 30 minutes on ice. After 24 hours the embryos were divided into groups according to their gfp signal intensity. Above the columns the calculated average gfp intensity in percent is indicated (see text for calculation). Similarly the results for mixed PNAs are shown in (**F**). Injections and evaluations were performed as in (**E**), except that 20 ng/μl mRNA were used. Note that the increased amount of mRNA results in higher numbers of average gfp intensity for comparable antisense function.

In order to establish conditions for the application of pePNA molecules for antisense approaches we established an assay for translation blocking. Cell culture based assays include the problem of the transport of PNAs (or PNA/DNA duplexes) into the cells [23]. To use an unbiased strategy we therefore used injection into fish embryos. The pePNAs were co-injected together with *gfp* mRNA into medaka embryos at the one-cell stage. As target sequence within the *gfp* mRNA we selected a region directly after the AUG (see Figure 2), which previously had been used for knock down strategies with morpholino oligos [9]. The embryos continued development and 24 hours later the gfp signal was observed under the fluorescence microscope. According to the gfp expression level, the embryos were categorized into 4 groups with strong, moderate, weak and no gfp signal, respectively (Figure 3A-D). To consider the small variations of the injection procedure, we calculated an overall number of gfp signal intensity for all embryos, which is based on different weights for the individual groups of embryos. For this calculation, strong embryos were weighted with 100%, embryos of the moderate group with 30%, those of the weak group with 10% and those showing no gfp signal were counted with 0%. From this an average number for all embryos was derived and then corrected by those for the injection of mRNA alone. The resulting value represents the average gfp intensity in % (mRNA alone results in 100%).

We first compared the efficiency of different lengths of the oligomers (12mer, 14mer, 16mer and 17mer; for sequence information see Figure 2). The pePNAs (200 μM) were co-injected with 10 ng/μl *gfp* mRNA. Although all PNA lengths were able to reduce the gfp signal in the embryos (Figure 3E and Additional file 2: Table S1), the strongest effect was observed for the 16mer PNA (Gfp16; reduction to 28% average gfp intensity).

To investigate the influence of the steric hindrance of the phosphonic ester residues, we synthesized a second set of 15 and 16mers. In this set an additional methylene group was introduced into the side chain (R-C3 instead of R-C2; see Figure 1) to elongate the alkyl spacer between the phosphonic esters and the oligomer backbone. Furthermore, the number of side chains was reduced, forming a hybrid oligomer of pe- and aegPNA components. We hypothesised an increased binding efficiency of these mixed PNAs and indeed found a high melting point (66.9°C) for Gfp16mix with complementary DNA. On the contrary a corresponding pure pePNA (Gfp17) showed a considerably lower melting point of 57.8°C (although containing one additional nucleotide compared to Gfp16mix). The presence of the aegPNA monomers in the molecules thus increased the binding affinity and this consequently resulted in a stronger reduction of the gfp signal compared to C2-pePNAs (data not shown). We therefore increased the amount of *gfp* mRNA for the co-injection assay to 20 ng/μl. This resulted in stronger gfp intensity, which allowed us to better evaluate the improved antisense effect of the mixed PNAs. At a concentration of 200 μM, a 15mer PNA with a mixed backbone (Gfp15mix) showed a reduction to 59% of the gfp fluorescence intensity. This result could be improved when a 16mer mixed PNA (Gfp16mix) was injected (41%), demonstrating a superior effect of 16mer PNAs also for mixed backbones. Similar effects were observed for a mixed PNA containing 4 TML residues (Gfp16mixL4). At higher concentrations of 400 μM or 600 μM the average gfp intensity was reduced to minimum levels of 12%. As a control for the specificity of the PNAs, we used a reference PNA with a completely unrelated sequence (Ref15mix). The gfp level in this control experiment was similar to the group of injected embryos without adding PNAs (Figure 3F). Furthermore, we did not observe any toxicity of the PNAs in the fish embryos at higher concentrations (up to 600 μM; see death rates in Additional file 3: Table S2).

### Uniform distribution of PNA molecules in medaka embryos

To get more information about the fate of the PNAs in the embryos, we injected a sulforhodamine labeled mixed PNA variant (Gfp16mixRho; for sequence information see Figure 2). After injection at the one-cell stage the PNA distributed equally into the dividing blastomeres (Additional file 4: Figure S2). A uniform distribution of the PNA in the embryo could also be observed at later stages, no signals were detected in the yolk. Using this labeled PNA we were able to exactly quantify the injection volume and the final concentration of the PNAs in the embryo. As a reference we used small beads (average 730 μm), which were soaked for several days with a defined concentration of the PNA. A comparison of the fluorescence signals of these beads with the injected embryos allowed us to determine the injection volume to 11.3 nl on average. Injection of antisense molecules at 100 μM consequently resulted in a concentration of 25 μM in the embryonic cells (the volume of the embryo was determined at the four-cell stage at 33 nl, for calculations see Methods section). Here uniform distribution within the cells was assumed.

### Knock down of the *six3* gene in medaka embryos by mixed PNAs

The Gfp-PNA molecules were tested in an *in vivo* setting, but the mixing of gfp-antisense PNAs with the *gfp* mRNA in the injection solution could lead to PNA/RNA hybridization prior to injection into the embryos. To avoid this possibility, we decided to test the targeting specificity of the PNAs in a complex *in vivo* environment, with a large excess of non-target mRNA. We therefore selected the endogenous *six3* gene as a target for PNA knock down experiments.

Six3 is part of the Six family of proteins, whose members are characterized by the presence of an N-terminal Six domain and a homeodomain [24]. Six genes are highly conserved across the animal kingdom. In *Drosophila* the *six3* homologue *optix* fulfills important functions in the development of the visual system [25]. In vertebrates, *six3* and *six6* are expressed in developing areas of the lens, neural retina, retinal-pigmented epithelium, nasal placodes, optic chiasm and forebrain. They take over important tasks in forming the rostral brain and the eye, especially the retina and the lens [26-28]. The critical function in eye development could also be demonstrated in medaka where inactivation of *six3* by morpholino knock-down resulted in lack of forebrain and eye structures [9].

**Figure 4:**
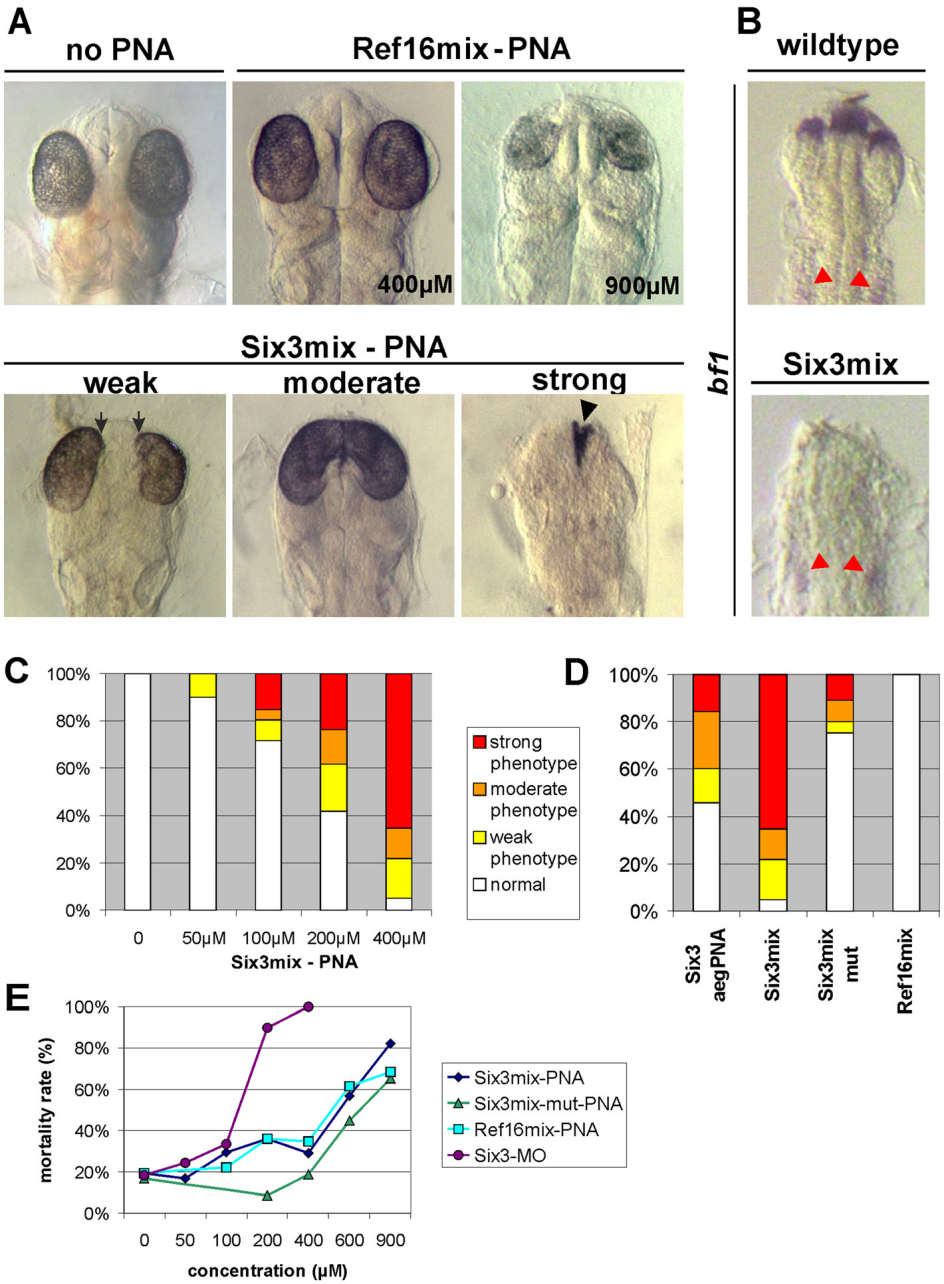
**Injection of medaka embryos with Six3-PNAs.** A wild-type embryo (no PNA injection) at 3 days is shown in (**A**). Embryos injected with 400 μM Ref16mix-PNA are indistinguishable from wild-type embryos; at 900 μM unspecific phenotypes were detected (both in A). The Six3mix-PNA injected embryos were evaluated 3 days after injection at stage 29. The phenotypes were divided into weak, moderate and strong (for criteria see text). Arrows indicate the anterior parts of the eyes pointing to the midline in weak phenotypes. The black arrowhead indicates remnants of the eye structures. All embryos in (**A**) are at stage 29 (34 somites). In (**B**) the expression of the *bf1* gene in wild-type and strongly affected embryos injected with Six3mix-PNAs is shown (both at stage 22; 9 somites). Note the presence of *bf1* expression in the otic vesicles, both in wild-type as well as in Six3mix-PNA injected embryos (marked by red arrowheads). Dorsal views of the anterior part of the embryos are shown. The quantitative evaluation of the *six3* knock down experiments is shown in (**C**) and (**D**) for the indicated PNA molecules. The percentages of phenotypes for the diagrams were calculated from the surviving embryos. PNAs shown in (**D**) were injected at 400 μM. (**E**) Shows the mortality rate of embryos injected at the indicated concentration (survival was examined 24 hrs after injection, see also Additional file 5: Table S3).

Based on the results of the gfp experiments we synthesized a 16mer mixed C3-PNA for the knock down experiments (referred to as Six3mix; for sequence information see Figure 2). The chosen sequence is a subset of a published morpholino 25mer directed against the *six3* 5’-region, directly upstream of the start codon [9]. After injection of the Six3mix-PNA a variety of eye and forebrain phenotypes were observed, in good agreement with previous results obtained for morpholino oligos in medaka embryos [9]. Based on the eye and forebrain phenotypes and in agreement with the literature [9] we divided these embryos into three groups. Embryos with a weak phenotype showed a size reduction of the eyes, which at the anterior part pointed towards the midline (Figure 4A arrows). Embryos with a cyclopic eye phenotype or strongly reduced eye size were characterized as moderate phenotype and embryos with almost no eye and forebrain formation were determined as strong phenotype (Figure 4A). The eye and forebrain phenotypes in Six3mix-PNA injected embryos were then confirmed by analyzing the expression of the winged-helix transcription factor *bf1*. It has been shown that *bf1* is essential for forebrain and eye development [29-31] and is regulated by Six3 [32]. As expected the *bf1* expression was strongly reduced in Six3mix-PNA injected embryos, especially in those with strong eye and forebrain phenotypes (Figure 4B).

The frequency of the obtained phenotypes was dependent on the Six3mix-PNA concentration. Whereas at lower concentrations (e.g. 50 μM) only 10% of the surviving embryos showed a weak phenotype, at higher concentrations (e.g. 400 μM) up to 95% of the surviving embryos exhibited eye and forebrain phenotypes (Figure 4A,C). A further increase of the PNA concentration to 900 μM only slightly improved the proportion of strong phenotypes, however, the frequency of mortality increased dramatically (82%; 51 of 62 injected embryos) indicating toxic effects of mixed PNAs at higher concentrations.

A critical question was how the introduced side chain modifications of the pePNA components affected the *in vivo* knock down efficiency compared to unmodified aegPNAs. Contrary to negative results reported for unmodified aegPNAs in fish embryos [19], we observed gene specific phenotypes in the injected embryos. However, mixed PNAs were more efficient than aegPNAs when compared directly (aeg- and mix-PNAs of the same length and sequence were used, both modified with TML at the C-teminus; see Figure 2). At 400 μM, where Six3mix-PNAs showed *six3* specific phenotypes in 95% of the surviving embryos, injection of Six3-aegPNAs resulted in only 54% affected embryos (Figure 4D and Additional file 5: Table S3). Furthermore, the fraction of strong phenotypes was largely reduced compared to Six3mix-PNAs (16% strong phenotypes for Six3-aegPNA versus 65% for Six3mix-PNA; calculated from Additional file 5: Table S3; see also Figure 4D). Finally, also the toxicity of aegPNAs was higher compared to mixed PNAs (compare death rates in Additional file 5: Table S3). At higher concentrations (600 μM) the results for the two PNA types was similar, however here the high death rate (>50%) indicates already considerable toxicity for both PNAs.

To examine the selectivity and the toxicity of the modified PNAs, we compared the frequency of the mortality and the obtained eye phenotypes in Six3mix-PNA injected embryos with two other control groups. First we injected a mixed PNA of the same length, but with a completely unrelated sequence (Ref16mix-PNA). Although the frequency of dead embryos slightly increased up to 400 μM PNA concentration, the surviving embryos did not show any malformations (Figure 4A; Ref16mix-PNA 400 μM). As a second control experiment to evaluate the selectivity of the antisense function we synthesized a 16mer mixed PNA with a single mismatch (Six3mix-mut-PNA; for sequence information see Figure 2). Although the mutant PNA also resulted in *six3* specific phenotypes, the frequency of embryos with eye and forebrain phenotype was strongly reduced (Figure 4D and Additional file 5: Table S3), suggesting that a single point mutation strongly affects the target selection of the mixed PNAs. This property offers the possibility to design highly specific antisense molecules that are able to select between individual allelic sequences, differing by single point mutations.

We next compared the Six3mix-PNAs directly with morpholino oligos, which represent the standard antisense molecules used for gene specific knock down in fish embryos [8]. We used a published target sequence for the medaka *six3* gene [9], which overlaps with our PNA sequences (for sequence information see Figure 2). We carefully compared the phenotypes at different embryonic stages. Morphologically, Six3mix-PNA injected embryos were not distinguishable from those injected with the morpholino oligo (data not shown), but they already appeared at lower concentrations compared to the PNAs (between 50 and 100 μM; see Additional file 5: Table S3). The appearance of unspecific phenotypes is well established for high concentrations of morpholino oligos [33]. We observed such phenotypes at concentrations of 200 μM, which already result in a high mortality rate of 90% (see Figure 4E and Additional file 5: Table S3). Again these unspecific phenotypes were highly similar to those observed for high doses of PNAs (a typical embryo is shown in Figure 4A), suggesting similar unspecific effects of both antisense molecules in the central nervous system and the eyes. For both morpholino oligos and PNAs the unspecific phenotypes only appeared at concentrations which already cause high mortality rates (200 μM for morpholino oligos and 600-900 μM for mix-PNAs). In addition they were clearly distinguishable from the *six3*-specific phenotypes and also appeared in embryos injected with high amounts of the control PNA Ref16mix, these embryos however lacked any *six3* specific phenotypes. To further extend the comparison, we performed a series of in situ hybridisation experiments. No differences were detectable between morpholino and mix-PNA injected embryos (see Additional file 6: Figure S3). Therefore, both types of antisense molecules generate the same range of *six3*-specific phenotypes in medaka embryos and also lead to highly similar unspecific effects at high doses. Similar to previous observations [3], we saw a higher efficiency of morpholino oligos at low concentrations. Morpholino oligos show a lower affinity for RNA compared to PNAs of the same length [34]. Our modification of aegPNAs into mixed PNAs resulted in enhanced knock down efficiency and interestingly, also in reduced affinity (see melting point determinations above). Therefore properties different from the affinity must account for the high efficiency of morpholino oligos, however, also the toxicity of these molecules peaks at substantially lower concentrations compared to the mixed PNAs (see Figure 4E).

To further demonstrate the specificity of the *six3* knock down, we injected a completely unrelated 16mer mix-PNA, directed against a sequence downstream of the *six3* AUG (Six3mix2; see Figure 2). The efficiency of this PNA was weak, since no phenotypes were observed at lower concentrations, however at 600 μM the expected typical *six3*-phenotypes appeared (see Additional file 5: Table S3). Therefore, two independent mixed PNAs directed against the *six3* mRNA resulted in the same gene-specific phenotypes, which are indistinguishable from those caused by morpholino oligos. Finally we performed a rescue experiment. For this purpose we generated mRNA of the human *six3* gene, which does not contain the targeted sequences of the medaka *six3*. At higher concentrations (25 and 50 ng/μl), injection of this mRNA causes specific phenotypes (enlarged eyes and ectopic retina, data not shown), we therefore switched to lower concentrations of 5 and 10 ng/μl. In a control experiment co-injection of *gfp*-mRNA with 400 μM Six3mix-PNA resulted in the expected phenotypes (see Additional file 7: Figure S4). However co-injection of *hSix3*-mRNA strongly reduced the overall number of *six3*-phenotypes (from 52% for the control embryos to 14 and 11%, respectively). Furthermore, no strong and moderate phenotypes appeared in these embryos any more. Therefore, the rescue experiments further demonstrate the specificity of the *six3* knock down by the mixed PNAs.

## Conclusions

PNAs are highly efficient antisense molecules, which bind their mRNA target sequences with high affinity. However, some properties make their application *in vivo* difficult. Contrary to previous observations [19] we show here that unmodified aegPNAs can be used for the specific knock down of genes in living animals. However, the introduction of phosphonic ester side chains to the backbone improves the properties of the PNAs considerably. The increased hydrophilicity resulted in high solubility and the modified pePNAs worked efficiently in translational blocking of mRNA. The combination with aegPNAs in mixed molecules combined the favourable hydrophilic properties of the pePNAs with the superior binding affinity of aegPNAs. As a result, we could demonstrate an efficient and highly specific knock down of a single medaka gene *in vivo*.

## Methods

### PNA synthesis

PNA monomer building blocks are commercially available. Optical pure (R-configuration according to CIP rules) pePNA monomer building blocks were synthesized according to a route reported previously [35-37]. N^2^-Boc-N^6^, N^6^, N^6^-trimethyl-(L)-lysine iodide (TML) as building block was prepared as published by Chen and Benoiton [38]. A schematic presentation of the building blocks is shown in Additional file 1: Figure S1. Ethyl esters of phosphonic acid are highly stable under physiologic conditions and against esterases [39]. All PNAs were synthesized on a fully automated solid phase synthesizer (Multisynthec Syro) according to a protocol developed by Koch [40]. aegPNAs (Six3-aegPNA) were synthesized by Eurogentec.

The PNAs were dissolved in nuclease free water by repeatedly shaking and vortex ting. Finally they were gently sonicated for 2 minutes with repeated pulses. Subsequently the PNAs were divided into aliquots of 100 μl (2 mM final concentration) and kept at −80°C.

### Measurement of melting points

The melting points of PNA-DNA duplexes were determined in Dulbecco’s PBS (1x) without Ca & Mg with a Thermo Genesys 10s UV–vis Spectrometer and a heated cuvette (Haake F3 S water bath).

### Microinjection into medaka embryos

Embryos of the medaka Cab strain were used for all experiments. Stages were determined according to Iwamatsu [41]. For the gfp experiments, first mRNA of *gfp* was *in vitro* transcribed using the T7 High Yield Message Marker Kit (Ambion) according to the manufacturer's instructions. The injection solution containing *gfp* mRNA (10 or 20 ng/μl), PNAs (50-600 μM) and RiboLock RNase inhibitor (2units/μl; Fermentas) were mixed on ice and then injected into embryos at the one-cell stage. Injection of *six3* antisense molecules was done at concentrations of 50–1200 μM PNAs or morpholino oligos. After injection the embryos were incubated at normal conditions (1x ERM buffer at 27°C).

### Quantification of the PNA concentration in the embryos

Sulforhodamine labeled PNAs (Gfp16mixRho) were injected at concentrations of 50 μM, 100 μM, 500 μM and 900 μM at the one-cell stage. At the four-cell stage pictures were made under the fluorescence microscope at standardised exposure times (1 ms, 10 ms, 100 ms and 1000 ms) and quantified using the ImageJ programme (background values were subtracted from all samples and only intensities within a linear range were considered for the quantification). For comparison, cellulose sulphate beads with an average diameter of 730 μm were soaked several days with the labeled PNA, shortly washed and then treated the same way under the fluorescence microscope. The volume of the embryo at the four-cell stage was determined by measuring the average diameter of the blastomeres (250 μm) resulting in a volume of 33 nl for the embryo. Using the volumes of the beads and the embryos the internal concentration of the PNA in the embryos could be calculated and resulted in an average value of 25.7 μM for injections with 100 μM PNA (injections were performed in a reproducible manner). The average injection volume therefore was 11.3 nl. All experiments were done in 5 fold repetitions.

### Whole-mount in situ hybridization

Whole-mount in situ hybridization using DIG-labeled probes was performed as described previously [42].

## Competing interests

The authors declare that they have no competing interests.

## Authors’ contributions

SD and GJ performed the injections with six3 antisense molecules, NA and BB performed the gfp knock down experiments. BW, HB and TL synthesized the PNAs. SD, NA and BB drafted the manuscript. TC conceived of the study and participated in its design and was responsible for coordinating and writing the manuscript. All authors read and approved the final manuscript.

## Supplementary Material

Additional file 1 **Figure S1.** Building blocks used for the chemical synthesis. Click here for file

Additional file 2 **Table S1.** Optimisation of the PNA length. The names of the PNAs are explained in Figure 2. The embryos were injected with a mixture of 10 ng/μl gfp mRNA and 200 μM PNAs preincubated for 30 minutes on ice. After 24 hours the embryos were divided into groups according to their gfp signal intensity. The average gfp intensity of the surviving embryos was then calculated as described in the text. Click here for file

Additional file 3 **Table S2.** Antisense function of mixed PNAs on gfp mRNA. Injections and evaluations were performed as described in Table 1, except that 20 ng/μl mRNA was used. Note that the increased amount of mRNA results in higher numbers of average gfp intensity for comparable antisense function. Click here for file

Additional file 4 **Figure S2.** Distribution of mix-PNAs in the embryo. Medaka embryos injected with 100 μM solution of Gfp16mixRho are shown in the 2-cell stage (A) and stage 24 (16 somites; B). A dashed line indicates the outline of the yolk. A lateral view of the embryo is shown in B. Click here for file

Additional file 5 **Table S3.***Six3* knock down by PNAs. PNAs and morpholino oligos were injected at the indicated concentrations and the embryos evaluated after 3 days at stage 29 according to the severity of the phenotype. “Phenotypes in surviving” indicates the percentage of surviving embryos showing *Six3* phenotypes. Click here for file

Additional file 6 **Figure S3.** Comparison of mixed PNA and morpholino phenotypes. Embryos were injected with 400 μM Six3mix-PNA (PNA) or 100 μM Six3-MO (MO) and at stage 20 analysed by in situ hybridisation with probes for *pax2* and *rx2.* Typical results are shown for each group of weak, moderate and strong phenotypes, wildtype (WT) embryos are shown as a reference. An arrowhead marks *pax2* expression in the mid-hindbrain boundary, note the reduced expression in the strong group. The eye vesicles in the weak group are smaller compared to wildtype embryos, in the moderate group their size is further reduced, in the strong group no eye vesicles were detectable. Click here for file

Additional file 7 **Figure S4.** Rescue of Six3mix-PNA injected embryos. Embryos were injected with 400 μM Six3mix-PNA (PNA) together with *gfp*-mRNA (10 ng/μl) or *hSix3*-mRNA (5 and 10 ng/μl). The phenotypes were then determined according to criteria described in the text. “Phenotypes in surviving” indicates the percentage of surviving embryos showing *six3* phenotypes. Click here for file
