# Supplementary figures and images for: Side chain modified peptide nucleic acids (PNA) for knock-down of six3 in medaka embryos
Source: BMC Biotechnol. 2012 Aug 17;12:50. doi: 10.1186/1472-6750-12-50 (PMC3469332; doi:10.1186/1472-6750-12-50)

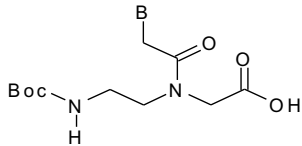

PNA

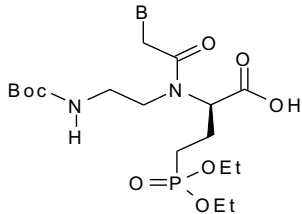

C2

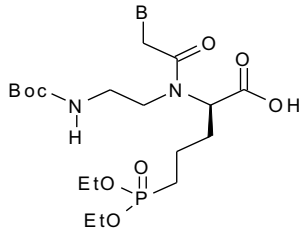

C3

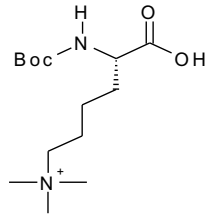

TML

Supplement: Additional file 1 — Figure S1. Building blocks used for the chemical synthesis. [file 1472-6750-12-50-S1.pdf]

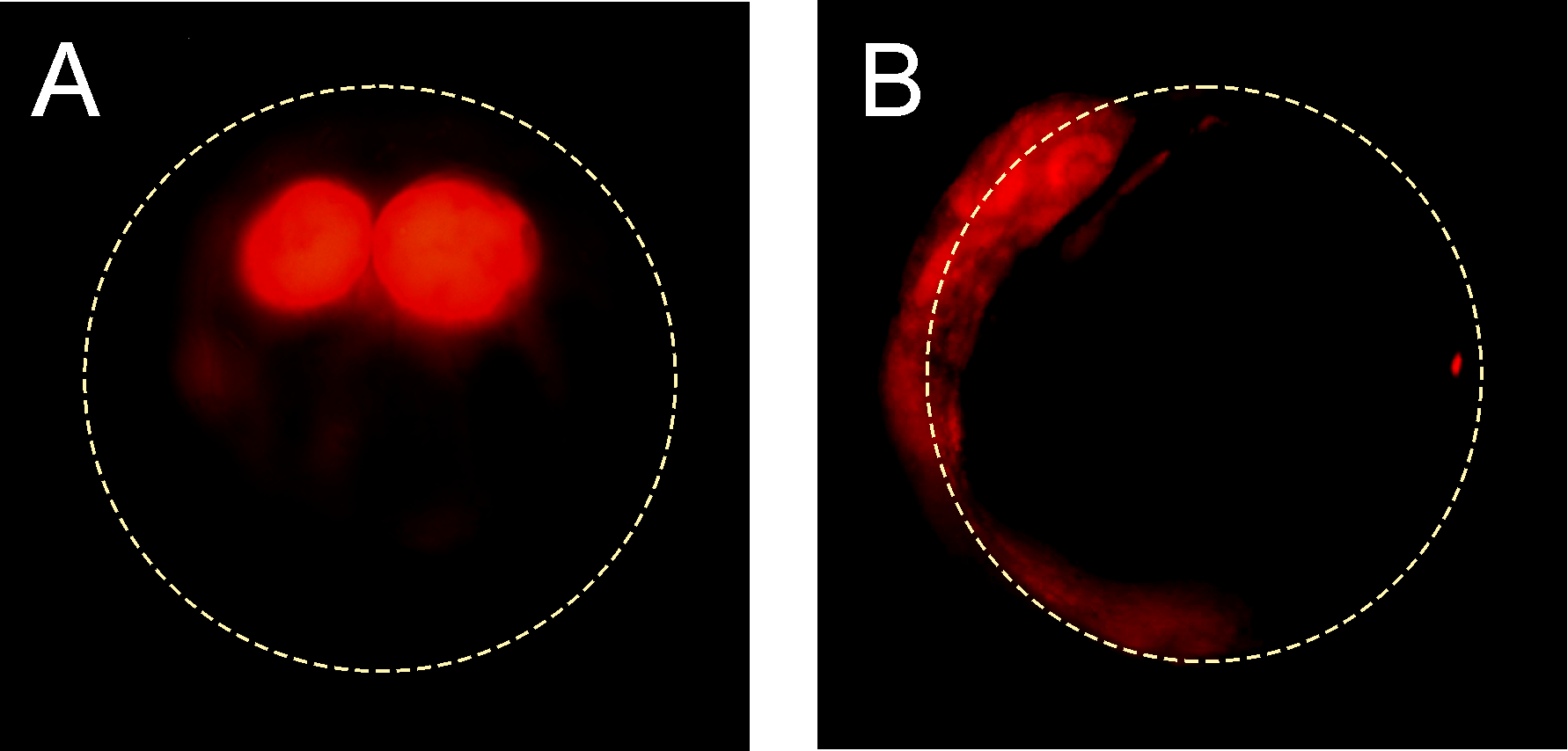

Supplement: Additional file 4 — Figure S2. Distribution of mix-PNAs in the embryo. Medaka embryos injected with 100 μM solution of Gfp16mixRho are shown in the 2-cell stage (A) and stage 24 (16 somites; B). A dashed line indicates the outline of the yolk. A lateral view of the embryo is shown in B. [file 1472-6750-12-50-S4.tiff]

Fig. S3

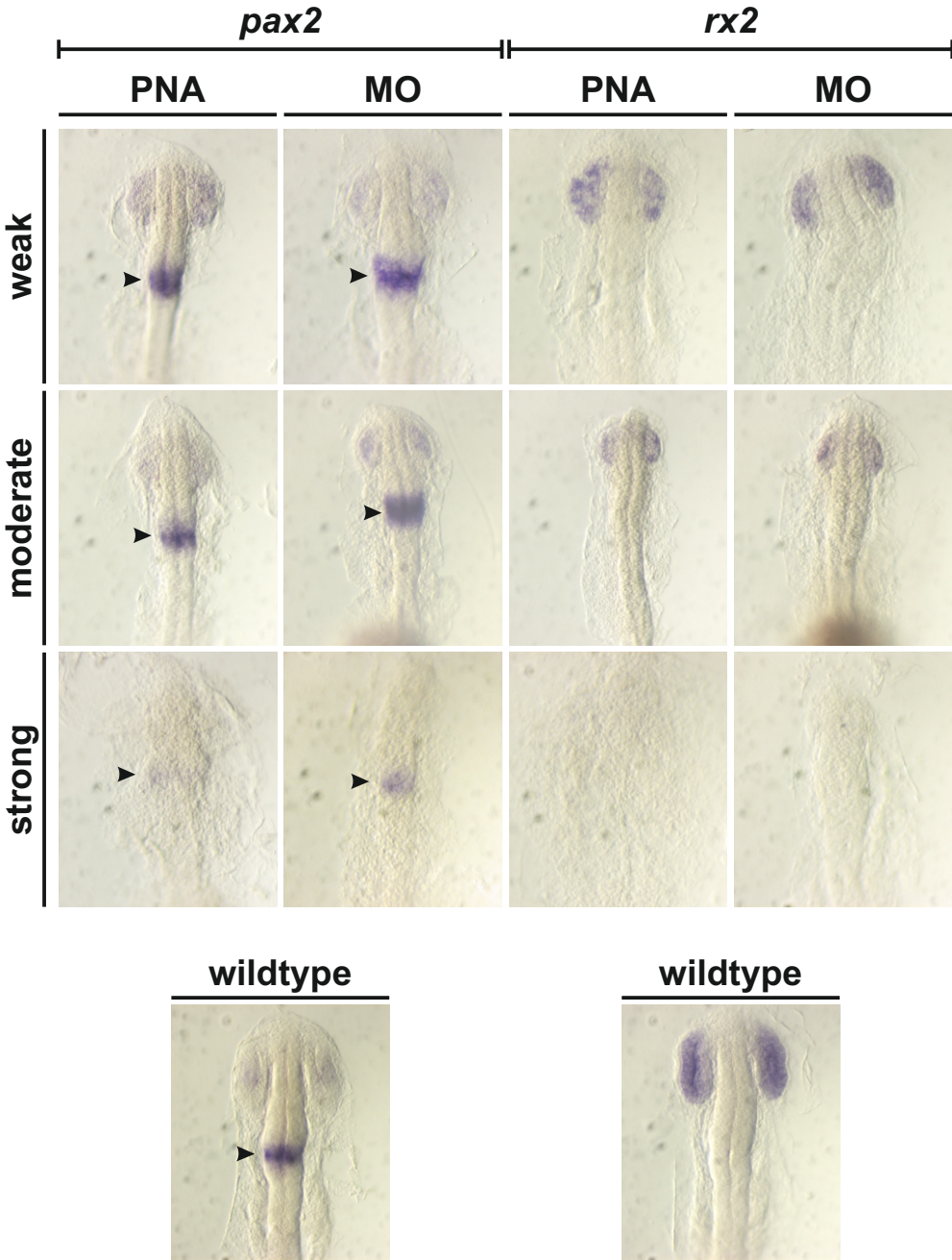

Supplement: Additional file 6 — Figure S3. Comparison of mixed PNA and morpholino phenotypes. Embryos were injected with 400 μM Six3mix-PNA (PNA) or 100 μM Six3-MO (MO) and at stage 20 analysed by in situ hybridisation with probes for pax2 and rx2. Typical results are shown for each group of weak, moderate and strong phenotypes, wildtype (WT) embryos are shown as a reference. An arrowhead marks pax2 expression in the mid-hindbrain boundary, note the reduced expression in the strong group. The eye vesicles in the weak group are smaller compared to wildtype embryos, in the moderate group their size is further reduced, in the strong group no eye vesicles were detectable. [file 1472-6750-12-50-S6.pdf]
