# Supplementary material for: Side chain modified peptide nucleic acids (PNA) for knock-down of six3 in medaka embryos
Source: BMC Biotechnol. 2012 Aug 17;12:50. doi: 10.1186/1472-6750-12-50 (PMC3469332; doi:10.1186/1472-6750-12-50)
Supplement: Additional file 2 — Table S1. Optimisation of the PNA length. The names of the PNAs are explained in Figure 2. The embryos were injected with a mixture of 10 ng/μl gfp mRNA and 200 μM PNAs preincubated for 30 minutes on ice. After 24 hours the embryos were divided into groups according to their gfp signal intensity. The average gfp intensity of the surviving embryos was then calculated as described in the text. [file 1472-6750-12-50-S2.pdf]

coinjected PNA	no PNA	Gfp12	Gfp14	Gfp16	Gfp17
injected embryos	72	71	71	71	67
dead	11	7	10	5	6
death rate	15%	10%	14%	7%	9%
gfp signal strong	50	31	19	5	32
gfp signal moderate	8	26	30	28	24
gfp signal weak	3	7	11	28	3
no gfp signal	0	0	1	5	2
average gfp intensity	100%	71%	55%	28%	75%
