# Supplementary material for: Side chain modified peptide nucleic acids (PNA) for knock-down of six3 in medaka embryos
Source: BMC Biotechnol. 2012 Aug 17;12:50. doi: 10.1186/1472-6750-12-50 (PMC3469332; doi:10.1186/1472-6750-12-50)
Supplement: Additional file 3 — Table S2. Antisense function of mixed PNAs on gfp mRNA. Injections and evaluations were performed as described in Table 1, except that 20 ng/μl mRNA was used. Note that the increased amount of mRNA results in higher numbers of average gfp intensity for comparable antisense function. [file 1472-6750-12-50-S3.pdf]

coinjected PNA	no PNA	Gfp15 mix	Ref15 mix	Gfp16 mix	Gfp16mixL4			
concentration		200µM	200µM	200µM	100µM	200µM	400µM	600µM
injected embryos	195	47	138	48	29	157	44	32
dead	22	2	15	4	3	18	6	2
death rate	11%	4%	11%	8%	10%	11%	14%	6%
gfp signal strong	129	13	96	6	10	8	0	0
gfp signal moderate	34	26	22	24	14	66	5	0
gfp signal weak	10	6	5	14	2	63	30	28
no gfp signal	0	0	0	0	0	2	3	2
average gfp intensity*	100%	59%	103%	41%	68%	30%	15%	12%
