# Supplementary material for: Side chain modified peptide nucleic acids (PNA) for knock-down of six3 in medaka embryos
Source: BMC Biotechnol. 2012 Aug 17;12:50. doi: 10.1186/1472-6750-12-50 (PMC3469332; doi:10.1186/1472-6750-12-50)
Supplement: Additional file 5 — Table S3.Six3 knock down by PNAs. PNAs and morpholino oligos were injected at the indicated concentrations and the embryos evaluated after 3 days at stage 29 according to the severity of the phenotype. “Phenotypes in surviving” indicates the percentage of surviving embryos showing Six3 phenotypes. [file 1472-6750-12-50-S5.pdf]

concentration	0	50µM	100µM	200µM	400µM	600µM	900µM
**Six3mix-PNA**							
number of embryos	83	36	65	86	142	67	62
dead	16	6	19	31	41	38	51
death rate	19%	17%	29%	36%	29%	57%	82%
strong phenotype	0	0	7	13	66	12	8
medium phenotype	0	0	2	8	13	7	2
weak phenotype	0	3	4	11	17	4	0
normal	67	27	33	23	5	6	1
phenotypes in surviv.	0%	10%	28%	58%	95%	79%	91%
**Six3mix2-PNA**							
number of embryos	83				21	31	50
dead	16				6	18	35
death rate	19%				29%	58%	70%
strong phenotype	0				0	5	4
medium phenotype	0				0	4	3
weak phenotype	0				0	3	0
normal	67				15	1	8
phenotypes in surviv.	0%				0%	92%	47%
**Six3-aegPNA**							
number of embryos	106		68	40	142	129	
dead	11		12	18	72	70	
death rate	10%		18%	45%	51%	54%	
strong phenotype	0		0	2	11	12	
medium phenotype	0		3	8	17	23	
weak phenotype	0		5	5	10	9	
normal	95		48	7	32	16	
phenotypes in surviv.	0%		14%	68%	54%	73%	
**Six3mixmut-PNA**							
number of embryos	96			104	124	105	112
dead	16			9	23	47	73
death rate	17%			9%	19%	45%	65%
strong phenotype	0			6	11	10	14
medium phenotype	0			7	9	5	1
weak phenotype	0			6	5	5	2
normal	80			76	76	38	22
phenotypes in surviv.	0%			20%	25%	34%	44%
**Ref16mix-PNA**							
number of embryos	83		27	25	26	39	19
dead	16		6	9	9	24	13
death rate	19%		22%	36%	35%	62%	68%
strong phenotype	0		0	0	0	0	0
medium phenotype	0		0	0	0	0	0
weak phenotype	0		0	0	0	0	0
normal	67		21	16	17	15	6
phenotypes in surviv.	0%		0%	0%	0%	0%	0%
**Six3-MO**							
number of embryos	66	29	27	59	17		
dead	12	7	9	53	17		
death rate	18%	24%	33%	90%	100%		
strong phenotype	0	12	11	6	0		
medium phenotype	0	6	7	0	0		
weak phenotype	0	2	0	0	0		
normal	54	2	0	0	0		
phenotypes in surviv.	0%	91%	100%	100%			
