# Supplementary material for: Side chain modified peptide nucleic acids (PNA) for knock-down of six3 in medaka embryos
Source: BMC Biotechnol. 2012 Aug 17;12:50. doi: 10.1186/1472-6750-12-50 (PMC3469332; doi:10.1186/1472-6750-12-50)
Supplement: Additional file 7 — Figure S4. Rescue of Six3mix-PNA injected embryos. Embryos were injected with 400 μM Six3mix-PNA (PNA) together with gfp-mRNA (10 ng/μl) or hSix3-mRNA (5 and 10 ng/μl). The phenotypes were then determined according to criteria described in the text. “Phenotypes in surviving” indicates the percentage of surviving embryos showing six3 phenotypes. [file 1472-6750-12-50-S7.pdf]

Fig. S4

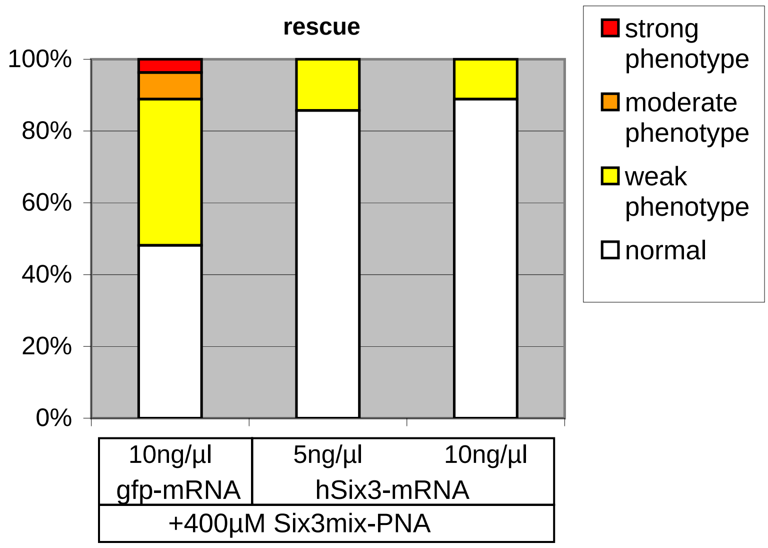

	Six3mix-PNA +		
	gfp-mRNA	hSix3 mRNA	
	10ng/µl	5ng/µl	10ng/µl
number of embryos	39	20	39
dead	12	6	12
death rate	31%	30%	31%
strong phenotype	1	0	0
moderate phenotype	2	0	0
weak phenotype	11	2	3
normal	13	12	24
phenotype in surviv.	52%	14%	11%
